# Acceptability of Interventions to Address Polypharmacy in Older Adult Outpatients: A Systematic Review and Meta‐Analysis

**DOI:** 10.1002/hsr2.70981

**Published:** 2025-07-31

**Authors:** Nav Persaud, Amal Rizvi, Aine Workentin, Becky Skidmore, Emily G. McDonald

**Affiliations:** ^1^ MAP Centre for Urban Health Solutions, Li Ka Shing Knowledge Institute, St. Michael's Hospital Unity Health Toronto Toronto Ontario Canada; ^2^ Department of Family and Community Medicine University of Toronto Toronto Ontario Canada; ^3^ Department of Family and Community Medicine St Michael's Hospital Toronto Ontario Canada; ^4^ Skidmore Research & Information Consulting Ottawa Ontario Canada; ^5^ Canadian Medication Appropriateness and Deprescribing Network Montreal Quebec Canada; ^6^ Division of General Internal Medicine, Department of Medicine McGill University Health Centre Montreal Quebec Canada

**Keywords:** acceptability, inappropriate prescribing, polypharmacy

## Abstract

**Background and Aims:**

Interventions to address potentially inappropriate prescribing (PIP), where risks outweigh benefits, are effective but often not implemented due to barriers (e.g., patient, provider, systems). Concerns about questioning healthcare providers or symptom resurgence when discontinuing medications may make PIP interventions less acceptable. This systematic review aims to determine the acceptability of PIP interventions among older adult outpatients.

**Methods:**

We searched MEDLINE, Embase, and other databases for controlled studies of PIP interventions including older adults (≥ 65 years) residing in community or care home settings. The review included interventions aimed at reducing PIP, whether clinical or external providers. We assessed risk of bias and performed a meta‐analysis.

**Results:**

Nine studies (*n* = 4,843) were included: six randomized controlled trials, two prospective cohort studies, and one pre‐post study. Studies spanned the US, England, Ireland, Lebanon, the Netherlands, Spain, and Switzerland. Seven out of nine (78%) studies were assessed as having a low risk of bias; two out of nine (22%) at moderate risk. Meta‐analysis showed no significant difference in patient satisfaction between PIP interventions and standard care, though satisfaction was slightly higher with PIP interventions (SMD 0.45; 95% CI −0.14 to 1.04, I² = 96%, *n* = 4,414). Meta‐analysis showed more patients discussed discontinuing medications with their prescriber after a PIP intervention (RR 4.32; 95% CI 0.0 to 56,270, I² = 43%, *n* = 429).

**Conclusion:**

PIP interventions are as acceptable to patients as usual care, despite some burden for patients and prescribers. Patients are more willing to engage in deprescribing conversations when a deprescribing intervention is present.

## Summary

**What is Already Known About This Subject:**

- Interventions to address potentially inappropriate prescribing (PIP) are effective but often underutilized due to patient, provider, and system barriers, including concerns about questioning providers or symptom resurgence from discontinuing medications.

**What This Study Adds:**

- This study highlights the importance of acceptability in implementing interventions addressing PIP, particularly for older adults at risk from polypharmacy. By encouraging healthcare providers to integrate these interventions into routine practice and fostering critical discussions about medication management, such efforts can reduce hospitalizations and improve health outcomes for vulnerable populations.

## 1 Introduction

Interventions to address potentially inappropriate prescribing (PIP), that by definition carries risks outweighing benefits [1], are effective but often not implemented because of a number of barriers [2, 3]. Although people are eager to reduce the number of medications taken [4, 5], patients may be reluctant to stop medicines they have been prescribed by a trusted provider and have been taking for a long time, and they may be unfamiliar with how to safely stop taking a medication. Patient preferences, including perceived necessity of medications and concerns about withdrawal or disease recurrence, play a critical role in deprescribing decisions [6]. Several studies have found that patients are reluctant to stop long‐term medications due to concerns about symptom recurrence, loss of benefit, or harming their relationship with a trusted provider [7, 8]. Qualitative and systematic reviews have identified common barriers including fear of health deterioration, uncertainty about how to deprescribe safely, and a desire to avoid challenging the prescriber's authority. In a qualitative study conducted among patients and providers in Malaysia, patients felt afraid to stop taking medications due to not wanting to “rock the boat” [9], and a systematic review found that fear of recurrence or worsening of a condition was a major barrier to deprescribing among patients taking cardiovascular medications [10]. A lack of trust between providers and patients can also be a barrier to deprescribing [2, 7, 8]. Deprescribing medicines requires clinicians to sometimes counter recommendations or orders made by colleagues and can require time to discuss with patients and expertise different from that required to start a medication [3]. Some PIP interventions involve community pharmacists or other professionals that may be unavailable or inaccessible in practice. PIP interventions also sometimes involve software that can be difficult to integrate with the electronic health record, can lead to alert fatigue, and can return suggested changes that are not implemented.

Studies without a control group report that PIP interventions have good reach (i.e., they can be implemented for most patients despite any technical or information technology issues), that suggested changes in medicines are often implemented, that interventions are acceptable to many patients, and can be implemented with good fidelity (i.e., close to how they were designed or intended to be implemented) [11]. Yet, PIP interventions are often not implemented and many suggested discontinuations are not made. This may be because implementing a PIP intervention requires additional time (e.g., a provider barrier) and may be associated with concerns and risks when compared with simply continuing prescriptions (e.g., both a patient and provider barrier). Implementing PIP interventions may be less acceptable than usual care.

This systematic review of controlled studies aims to evaluate the acceptability of interventions addressing PIP in older adult outpatients. This systematic review was conducted to inform a clinical practice guideline related to PIP interventions and it complements another systematic review that focuses on the effectiveness of PIP interventions. We recognize that there are many factors beyond acceptability that determine whether or not these interventions are implemented.

## 2 Methods

The protocol for this systematic review was developed by the University of Ottawa's Evidence Review and Synthesis Centre as part of the work of the Canadian Task Force on Preventive Health Care [12]. The systematic review was registered (PROSPERO CRD42022302313 and Open Science Framework, https://osf.io/urj4b/). The results are reported in accordance with Preferred Reporting Items for Systematic reviews and Meta‐Analyses (PRISMA) guidance (see Appendix for PRISMA checklist) [13]. We separately report the results of a systematic review related to the effectiveness of the interventions.

### 2.1 Eligibility

We included controlled studies of PIP interventions that included older adults, aged 65 years or older, residing in the community, long‐term care homes, or nursing homes. We included studies of any intervention aimed at reducing PIP, including reviews by health care providers who were part of the clinical team or independent from the clinical team. Eligible comparators included usual care or minimal interventions such as generic reminders about safe prescribing. We included studies that contrasted acceptability outcomes between the PIP intervention group and a control group.

We excluded studies that solely enrolled adults younger than 65 years of age and studies that enrolled only inpatients. We excluded studies that addressed only the prescribing of antibiotics as antibiotic stewardship is a topic with its own literature (see eTable 1 for examples).

### 2.2 Information Sources and Search Strategy

An experienced medical information specialist (BS) developed the search strategy in consultation with the review team. The MEDLINE strategy was peer‐reviewed by another senior information specialist using the PRESS checklist [14]. Using the multifile option and deduplication tool within Ovid, we searched Ovid MEDLINE® ALL, Embase Classic+Embase, and APA PsycInfo. We also searched the Cochrane Database of Systematic Reviews (Wiley version). We utilized a combination of controlled vocabulary (e.g., “Polypharmacy”, “Health Services for the Aged”, “Patient Acceptance of Health Care”) and keywords (e.g., “multiple prescriptions”, “older adult”, “personal choice”) in the searches, adjusting vocabulary and syntax as necessary across the databases. We limited results to French or English language and the publication years 2004 to the present. Where possible, we removed animal‐only records. We downloaded and deduplicated the records using EndNote version 9.3.3 (Clarivate Analytics).

### 2.3 Study Selection

Following completion of a pilot exercise, titles and abstracts were screened by two independent reviewers (AR, AW) by applying the inclusion and exclusion criteria. Disagreements were resolved through discussion or by consulting with a third reviewer (NP). Full‐text articles were assessed against the inclusion and exclusion criteria by two independent reviewers (AR, AW) and disagreements were resolved through discussion.

### 2.4 Data Abstraction and Risk of Bias Assessment

We (AR, AW, NP) developed and piloted a data abstraction tool that recorded information about the characteristics of each study, details about the intervention and relevant outcomes. Two reviewers (AR, AW) extracted information about each study. We used the Cochrane revised risk of bias instrument for randomized controlled trials, also known as RoB 2 tool, to assess randomized controlled trials and the Risk of Bias in non‐randomized Studies of Interventions or ROBINS‐I tool for observational studies [15, 16]. Both RoB 2 and ROBINS‐1 evaluate bias across domains such as selection of participants, classification of interventions, missing data, randomization (for randomized studies), and confounding (for non‐randomized studies). For each domain, we then categorized studies as being low risk, high risk, or having some concerns. Based on the domain rankings, each study was then given an overall risk of bias classification of low risk, high risk, or some concerns.

### 2.5 Synthesis of Included Studies

We statistically pooled results in a meta‐analysis using a random effects model using the inverse variance method if two or more studies were similar in terms of participants and outcomes. Results were expressed as risk ratios (RR) for dichotomous outcomes and as standardised mean differences (SMD) for continuous outcomes. For cluster randomized controlled trials, we adjusted events and sample sizes based on the design effect that considers the cluster size and intra‐cluster correlation [17]. We assessed statistical heterogeneity using the restricted maximum likelihood inconsistency or I^2^ measure [18]. To try to explain statistical heterogeneity, we performed subgroup analyses. We report results of the vote counting method based on the direction of the effect [19]. We assessed publication bias using funnel plots and Egger's regression test [20]. Meta‐analysis was performed using Meta‐Mar (version 3.5.1, 2018). For patient satisfaction, we pooled data from studies that used different instruments by converting mean or median scores to percentages using the maximum possible score, and we converted proportions of patients rating their satisfaction above a certain threshold to a percentage, before estimating the standardized mean difference (SMD) using a random effects model. We employed a random effects model rather than a fixed model on the assumption that the studies in different settings and clinical contexts could be measuring different true effects. For the proportion of patients who discussed medication discontinuation, we estimated the risk ratio (RR) using a random effects model.

## 3 Results

**Figure 1:**
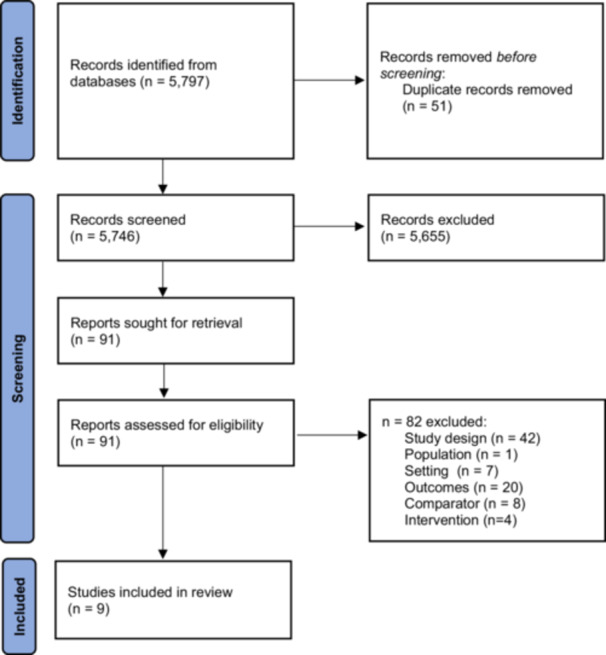
PRISMA flow diagram of controlled studies assessing acceptability of PIP interventions.

Our search, ran on June 18, 2024, returned 5746 unique records. After assessing 91 full‐text articles, we included nine studies (Figure 1).

The nine included studies are summarized in Table 1. There were six randomized controlled trials including four that randomized individual patients [21, 22, 23, 24] and two cluster trials [25, 26], two non‐randomized controlled trials [27, 28] (or prospective cohort studies) and one pre‐post study [29]. Two studies were conducted in each of the United States of America and England, and one in Ireland, Lebanon, Netherlands, Spain and Switzerland. Seven studies were based in primary care practices, one in a clinic for people with Alzheimer's disease and one in a community pharmacy. The interventions included medication reviews, educational programs for prescribers about deprescribing, and computer decision aids.

**Table 1:** Characteristics of controlled studies assessing acceptability of PIP intervention.

Study	Study design	Setting and population	Intervention type and description	Contrasted outcome
Alaa Eddine 2023	Nonrandomized controlled trial	Patients at a community healthcare facility, ≥ 65, taking ≥ 5 drugs who pick up their medications themselves, in Lebanon.	Education Program and Medication Review: Pharmacists reviewed medications using STOPP/START, Medscape, and guidelines, then made recommendations to physicians. The intervention group completed the MMPSS after, and the control group before, the intervention.	Patient satisfaction
Community Pharmacy Medicines Management Project Evaluation Team 2007	Randomized controlled trial	Primary care patients (≥ 17) with congenital heart disease, in England.	Medication Review: A community pharmacist conducted an initial consultation to assess therapy suitability, compliance, lifestyle, and social or support issues.	Patient satisfaction
Del Cura‐González 2022	Cluster randomized controlled trial	Primary care patients, age 65–74 with ≥ 3 chronic conditions, taking ≥ 5 drugs, who had made at ≥ 1 visit to GP in the past year, in Spain.	Education Program and Medication Review: GPs completed a 4‐week eMULTIPAP course, then conducted structured patient interviews, reviewed treatment plans, and developed pharmacological plans.	Patient satisfaction
Hamilton 2007	Randomized controlled trial	Unselected patients attending primary care appointments, in England.	Patients received a Self‐completed Agenda Form (SCAF) to fill out while waiting and gave to the GP at their appointment. Physicians could use the SCAF as a decision support tool at their discretion.	Patient satisfaction
Hughtenburg 2009	Nonrandomized controlled trial	Patients at community pharmacies successively discharged from the hospital taking ≥ 5 prescription drugs, in the Netherlands.	Medication review: Patients underwent an extensive medication review and drug counseling.	Patient satisfaction
Linksky 2022	Pre‐post study	Primary care or women's health clinic patients at risk of hypoglycemia from diabetes overtreatment or prescribed a proton pump inhibitor for 90 days, in the United States.	Education program: EMPOWER brochures were sent to eligible patients 2 weeks before their primary care visit to encourage medication discontinuation with provider guidance	Discussions about medication discontinuation
McCarthy 2022	Cluster randomized controlled trial	Patients at general practices age ≥ 65 with ≥ 15 regular medicines, in Ireland.	Education program and medication review: GPs accessed the SPPiRE website, completed an educational module, and used a template for medication reviews to identify PIP, deprescribing opportunities, and patient care priorities.	Patient satisfaction
Mecca 2022	Randomized controlled trial	Primary care patients, ≥ 65 prescribed ≥ 7 medications including at least one for hypertension and diabetes mellitus, in the United States.	Computerized decision support: The TRIM tool provided primary care physicians with individualized reports on PIMs, treatment deintensification, and adherence/cognition issues.	Discussions about medication discontinuation
Messerli 2018	Randomized controlled trial	Patients at an Alzheimer's Disease centre using ≥ 4 prescriptions, in Switzerland.	Medication review: Study pharmacists performed a polymedication checks on patients to identify drug‐related problems.	Patient satisfaction

Seven out of nine (78%) studies were assessed as having a low risk of bias, while two out of nine (22%) were assessed at moderate risk. The two studies with moderate risk were non‐randomized controlled trials. Reasons for risk of bias in these studies included bias in classification of interventions, measurement of outcomes, deviations from intended interventions, and confounding factors (see eFigure 1, eFigure 2, eFigure 3, eFigure 4 in the Appendix). There was no evidence of publication bias in the meta‐analyses described below.

**Figure 2:**
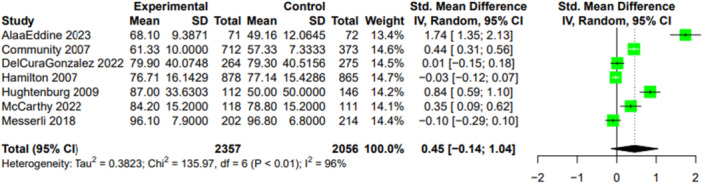
Meta‐analysis of patient satisfaction with potentially inappropriate prescribing interventions versus usual care among older adult outpatients.

Patient satisfaction was reported in seven studies, of which five reported scores for the intervention and control group using different types of instruments and two reported the proportions in each group rating their satisfaction above a certain score. Of the seven studies reporting satisfaction, five reported greater satisfaction in the intervention arm and two reported greater satisfaction in the usual care arm. Meta‐analysis showed no statistically significant difference in patient satisfaction between groups with the point estimate consistent with slightly higher satisfaction with PIP interventions (SMD 0.45; 95% CI −0.14 to 1.04, I^2^ 96%, *n* = 4,414) (see Figure 2). Subgroup analyses did not yield any substantially different results.

**Figure 3:**
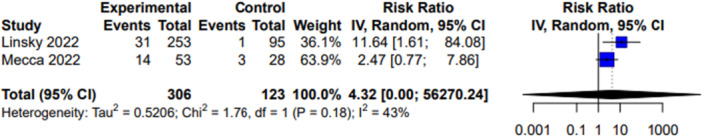
Meta‐analysis of discussions about medication discontinuation among older adult outpatients receiving potentially inappropriate prescribing interventions versus usual care.

The two studies that did not report patient satisfaction reported the proportion of patients who had a discussion about discontinuing medications with their prescriber. These discussions about deprescribing may indirectly be a measure of patient satisfaction, especially in the one study where patients needed to initiate the discussion after receiving a brochure in the mail [19]. Both studies found discussions were more likely with the PIP intervention. A pre‐post study found a greater likelihood of discussions about potentially inappropriate medications after patients received a brochure about deprescribing [19]. A trial of a tailored report with suggested medication changes found more discussion of medication changes [20]. Meta‐analysis of the proportion who had a discussion indicated more patients having discussions with PIP interventions (RR 4.32, 95% CI 0.0 to 56270, I^2^ = 43, *n* = 429) (see Figure 3).

Potential publication bias was assessed using Egger's regression test and visual inspection of a funnel plot (see eFigure 5 in the Appendix). While the funnel plot appeared asymmetric due to one outlying study, Egger's test did not indicate statistically significant asymmetry (intercept = 5.82, *p*  =  0.406). Given the small number of studies (*n* =  7), the test may have been underpowered to detect bias.

## 4 Discussion

This systematic review of controlled studies found that PIP interventions are at least as acceptable to patients based on patient reports of satisfaction and documented discussions with patients about deprescribing. This finding is notable because PIP interventions might be expected to be less acceptable to patients given that they involve extra time, potentially difficult discussions with a prescriber and risks associated with discontinuing medications. These findings can be combined with effects of PIP interventions on health outcomes to inform guidance on implementing PIP interventions [9]. The main implication of our findings is that the implementation of effective PIP interventions should be acceptable to patients. Thus, patient acceptability should not be viewed as a barrier to implementing PIP interventions.

Our findings are consistent with findings of qualitative studies of the acceptability of PIP interventions and with findings of clinical trials. Qualitative studies indicate that PIP interventions are generally acceptable to patients and providers and that the time requirements of interventions is an important determinant of acceptability [30, 31, 32, 33]. Noncontrolled quantitative studies have found that patients and providers generally accept suggested changes but may be more likely to implement changes under certain circumstances such as when patients are prescribed many medications and when reviews are done periodically [11]. Systematic reviews of PIP interventions indicate that they are effective at reducing inappropriate prescribing and that they may reduce hospitalizations and deaths, and that outpatient visits may be temporarily increased during the discontinuation of certain medications that cause withdrawal effects [34, 35, 36, 37].

This systematic review has several strengths and limitations. We included controlled studies that quantitatively compared the acceptability of PIP interventions to usual care. As far as we know, no other systematic review has focused on the acceptability of PIP interventions, although patients have been found to have positive attitudes toward deprescribing [38]. The included studies were designed to assess the effectiveness of the intervention and not its acceptability. The studies were not blinded, and outcomes based on patient reports such as satisfaction could be due to knowledge of the group of allocation rather than the PIP interventions, although we included controlled observational studies where this would have been less of an issue. We included discussions about medication discontinuations as an indirect measure of acceptability to patients but in one of the two studies patients may not have wanted these discussions, although in the other study, patients initiated the discussions. There was substantial statistical heterogeneity that was not easily explained by the characteristics of the studies, perhaps in part because different instruments were used to assess satisfaction and results were reported in different ways although subgroup analyses did not yield substantially different results. The findings based on nine studies, mostly set in Europe, may not be applicable to all medication types or all practice settings. Factors beyond acceptability also determine whether PIP interventions are implemented. While our review provides insights into the acceptability of these interventions, broader considerations are crucial for understanding their implementation in practice.

## 5 Conclusion

Interventions to address PIP are at least as acceptable to patients as usual care. When combined with qualitative acceptability studies and effectiveness studies, these results indicate that PIP interventions can be implemented safely and in ways that are acceptable to patients. Patient acceptability should not be a barrier to implementing PIP interventions.

## Author Contributions

**Nav Persaud:** conceptualization, formal analysis, investigation, supervision, visualization, writing – original draft, writing – review and editing. **Amal Rizvi:** data curation, methodology, project administration, writing – review and editing. **Aine Workentin:** data curation, methodology, project administration, writing – review and editing. **Becky Skidmore:** data curation, methodology, resources. **Emily G McDonald:** conceptualization, funding acquisition, supervision, visualization, writing – original draft, writing – review and editing.

## Conflicts of Interest

Dr Persaud reported grants from the Canadian Institutes of Health Research and the Canada Research Chairs program. Dr McDonald reported co‐ownership of MedSafer, a for‐profit business.

## 1 Transparency Statement

The lead author Nav Persaud affirms that this manuscript is an honest, accurate, and transparent account of the study being reported; that no important aspects of the study have been omitted; and that any discrepancies from the study as planned (and, if relevant, registered) have been explained.

## Supporting information

PIPacceptabilityAppendix2025 04 04.

PRISMA 2020 checklist.

## Data Availability

The data that support the findings of this study are available from the corresponding author upon reasonable request.
