# Supplementary material for: Acceptability of Interventions to Address Polypharmacy in Older Adult Outpatients: A Systematic Review and Meta‐Analysis
Source: Health Sci Rep. 2025 Jul 31;8(8):e70981. doi: 10.1002/hsr2.70981 (PMC12313817; doi:10.1002/hsr2.70981)

**Search details**

Ovid Multifile

Database: Embase Classic+Embase <1947 to 2024 June 17>, APA PsycInfo <1806 to June Week 2 2024>, Ovid MEDLINE(R) ALL <1946 to June 17, 2024>

Search Strategy:

--------------------------------------------------------------------------------

1 exp Polypharmacy/ (34598)

2 (polypharm* or poly-pharm*).tw,kw,kf. (40410)

3 (polymedic* or poly-medic*).tw,kw,kf. (2357)

4 (polyprescri* or poly-prescri*).tw,kw,kf. (42)

5 (polypragmas* or poly-pragmas*).tw,kw,kf. (341)

6 Deprescriptions/ (3320)

7 (deprescri* or de-prescri*).tw,kw,kf. (6719)

8 ((cancel* or ceas* or cessation? or discontinu* or halt* or stop* or terminat*) adj2 (medicat* or medicine? or prescrib* or prescription?)).tw,kw,kf. (25049)

9 ((cancel* or ceas* or cessation? or discontinu* or halt* or stop* or terminat*) adj (drug or drugs)).tw,kw,kf. (2341)

10 Inappropriate Prescribing/ (9912)

11 ((appropriate* or inappropriate* or incorrect* or indiscriminat* or unnecessar* or rational* or irrational* or optimal* or optimum or suboptim* or sub-optim*) adj2 (medicat* or medicine? or prescrib* or prescription? or OTC or "over-the-counter" or "behind-the-counter")).tw,kw,kf. (49867)

12 ((appropriate* or inappropriate* or incorrect* or indiscriminat* or unnecessar* or rational* or irrational* or optimal* or optimum or suboptim* or sub-optim*) adj (drug or drugs)).tw,kw,kf. (26362)

13 ((excess* or multipl* or "five or more" or "5 or more") adj2 (medicat* or medicine? or prescrib* or coprescrib* or co-prescrib* or prescription? or coprescription? or co-prescription? or OTC or "over-the-counter")).tw,kw,kf. (17674)

14 ((excess* or multipl* or "five or more" or "5 or more") adj (drug or drugs)).tw,kw,kf. (27845)

15 (many medication* or many medicine* or many drug?).tw,kw,kf. (19508)

16 ((concomitant* or concurrent*) adj2 (medicat* or medicine? or prescrib* or prescription? or OTC or "over-the-counter" or "behind-the-counter")).tw,kw,kf. (22220)

17 ((concomitant* or concurrent*) adj (drug or drugs)).tw,kw,kf. (5211)

18 ((omit* or omission?) adj2 (medicat* or medicine? or prescrib* or prescription? or drug or drugs)).tw,kw,kf. (2230)

19 ((under$2 or over$2) adj2 (medicat* or prescrib* or prescription?)).tw,kw,kf. (24318)

20 (underprescri* or overprescri*).tw,kw,kf. (4528)

21 (quality adj2 (prescrib* or prescription?)).tw,kw,kf. (3236)

22 ((multidrug? or multi-drug?) adj2 (pharmacotherap* or pharmaco-therap* or prescrib* or prescription? or regim* or therap* or treatment?)).tw,kw,kf. (15539)

23 (multiple adj (pharmacotherap* or pharmaco-therap*)).tw,kw,kf. (118)

24 ((medication? or medicine? or prescrib* or prescription?) adj3 cascad*).tw,kw,kf. (510)

25 ((medication? or medicine? or prescrib* or prescription?) adj3 continuum*).tw,kw,kf. (234)

26 ((medication? or medicine? or prescrib* or prescription?) adj3 legac*).tw,kw,kf. (180)

27 ((medication? or medicine? or prescrib* or prescription?) adj3 (multi* chronic* or multi* comorbid* or multi* co-morbid* or multimorbid* or multi-morbid*)).tw,kw,kf. (951)

28 (prescrib* adj3 (harm or harmed or harms or harming or harmful*)).tw,kw,kf. (559)

29 Potentially Inappropriate Medication List/ (3972)

30 STOPP.tw,kw,kf. (2123)

31 "STOPP/START".tw,kw,kf. (995)

32 ((Beers or McLeod or NPS) adj2 criteri*).tw,kw,kf. (3142)

33 "Fit fOR The Aged".tw,kw,kf. (401)

34 ((FORTA or RASP or Pricus) adj2 (criteri* or instrument? or list*)).tw,kw,kf. (120)

35 Assessing Care of Vulnerable Elderly.tw,kw,kf. (2)

36 ACOVE.tw,kw,kf. (225)

37 Medication Appropriateness Index.tw,kw,kf. (464)

38 Medication Regimen Complexity.tw,kw,kf. (691)

39 Prescribing Optimi#ation Method.tw,kw,kf. (6)

40 Systematic Tool to Reduce Inappropriate Prescribing.tw,kw,kf. (30)

41 (strip adj5 (medication? or medicine? or prescrib* or prescription?)).tw,kw,kf. (93)

42 or/1-41 [POLYPHARMACY] (281066)

43 Adult/ (15631144)

44 exp Aged/ (7575928)

45 aged.ti,kw,kf. (224805)

46 ((age? or year?) adj2 ("65" or "66" or "67" or "68" or "69" or "70" or "71" or "72" or "73" or "74" or "75" or "76" or "77" or "78" or "79" or "80" or "81" or "82" or "83" or "84" or "85" or "86" or "87" or "88" or "89" or "90" or "91" or "92" or "93" or "94" or "95" or "96" or "97" or "98" or "99" or "100")).tw,kw,kf. (2616645)

47 (elder? or elderly or geriatric* or gerontolog* or old-age? or senior?).tw,kw,kf. (1264581)

48 (older adj2 (adult* or female? or male? or man or men or patient? or person? or people? or population? or wom#n)).tw,kw,kf. (838790)

49 Retirement/ (37118)

50 (retiree? or retired or retirement).tw,kw,kf. (80426)

51 (boomer? or babyboomer* or baby-boomer*).tw,kw,kf. (6269)

52 Health Services for the Aged/ (56826)

53 Homes for the Aged/ (27994)

54 ((old age or nursing or longterm care or long-term care or LTC) adj (facilit* or home? or residen*)).tw,kw,kf. (134755)

55 or/43-54 [SENIOR FILTER] (20186596)

56 42 and 55 [POLYPHARMACY - SENIOR FILTER] (127721)

57 exp Animals/ not Humans/ (18205877)

58 56 not 57 [ANIMAL-ONLY REMOVED] (108475)

59 (comment or editorial or news or newspaper article).pt. (2556061)

60 (letter not (letter and randomized controlled trial)).pt. (2578336)

61 58 not (59 or 60) [OPINION PIECES REMOVED] (106693)

62 Choice Behavior/ (331139)

63 (choice? adj2 behavio?r*).tw,kw,kf. (16115)

64 Cooperative Behavior/ (88194)

65 Decision Making/ (499332)

66 Decision Making, Shared/ (19815)

67 decision aid*.tw,kw,kf. (13436)

68 ((guide? or guiding or make or making or makes or made or shar* or support*) adj2 (choice? or choos* or consent* or decid* or decision*)).tw,kw,kf. (876784)

69 Patient Education as Topic/ (199534)

70 Patient Acceptance of Health Care/ (126871)

71 Patient Participation/ (71091)

72 Patient Preference/ (39614)

73 Patient Satisfaction/ (276090)

74 ((engag* or involv* or participat*) adj3 (patient? or person$2 or personally or man or men or "man's" or "men's" or wom#n or "woman's" or "women's")).tw,kw,kf. (520093)

75 exp Patients/px [psychology] (18681)

76 Uncertainty/ (83882)

77 ((accept* or consider* or choice? or choos* or chose? or decid* or decis* or expect* or input* or knowledge* or opinion* or participat* or perspective? or prefer* or respons* or satisf* or uncertain* or understand* or willing*) adj2 (female? or male? or man or men or "man's" or "men's" or patient? or person$2 or personally or wom#n or "woman's" or "women's")).tw,kw,kf. (1306274)

78 ((analys#s or valuation? or value? or valuing) adj2 (conjoint or contingent)).tw,kw,kf. (6896)

79 (choice? adj1 (discrete or experiment*)).tw,kw,kf. (15618)

80 ((patient? or person$2 or personally or man or men or "man's" or "men's" or wom#n or "woman's" or "women's") adj (centered or centred or focus*)).tw,kw,kf. (147006)

81 ((patient* or person#2) adj priorit*).tw,kw,kf. (3925)

82 Informed Consent/ (185104)

83 (informed adj (choice* or choos* or consent* or decid* or decision*)).tw,kw,kf. (206947)

84 ((patient? or person$2 or personally or man or men or "man's" or "men's" or wom#n or "woman's" or "women's") adj2 consent*).tw,kw,kf. (51524)

85 ((adher* or nonadher* or non-adher* or inten* or refus* or reject* or uptake or willing*) adj2 (initiat* or intervention? or therap* or treat*)).tw,kw,kf. (389868)

86 (preference? adj1 (elicit* or reveal* or scor* or stated)).tw,kw,kf. (8963)

87 (trade off? or tradeoff? or trade-off?).tw,kw,kf. (105416)

88 Information Seeking Behavior/ (8463)

89 (inform* adj1 seek*).tw,kw,kf. (20840)

90 or/62-89 [PATIENT ACCEPTABILITY/DECISION-MAKING/PARTICIPATION] (4066230)

91 61 and 90 [POLYPHARMACY - SENIORS - PATIENT ACCEPTABILITY/DECISION-MAKING/PARTICIPATION] (18540)

92 limit 91 to english (17842)

93 limit 91 to french (130)

94 92 or 93 [LANGUAGE LIMITS APPLIED] (17950)

95 94 use medall [MEDLINE RECORDS] (6049)

96 exp *polypharmacy/ (10672)

97 (polypharm* or poly-pharm*).ti,kw,kf. (15343)

98 (polymedic* or poly-medic*).ti,kw,kf. (426)

99 (polyprescri* or poly-prescri*).ti,kw,kf. (8)

100 (polypragmas* or poly-pragmas*).ti,kw,kf. (83)

101 *deprescription/ (2242)

102 (deprescri* or de-prescri*).ti,kw,kf. (4092)

103 ((cancel* or ceas* or cessation? or discontinu* or halt* or stop* or terminat*) adj2 (medicat* or medicine? or prescrib* or prescription?)).ti,kw,kf. (1772)

104 ((cancel* or ceas* or cessation? or discontinu* or halt* or stop* or terminat*) adj (drug or drugs)).ti,kw,kf. (308)

105 exp *prescribing error/ (2219)

106 ((appropriate* or inappropriate* or incorrect* or indiscriminat* or unnecessar* or rational* or irrational* or optimal* or optimum or suboptim* or sub-optim*) adj2 (medicat* or medicine? or prescrib* or prescription? or OTC or "over-the-counter" or "behind-the-counter")).ti,kw,kf. (10660)

107 ((appropriate* or inappropriate* or incorrect* or indiscriminat* or unnecessar* or rational* or irrational* or optimal* or optimum or suboptim* or sub-optim*) adj (drug or drugs)).ti,kw,kf. (3629)

108 ((excess* or multipl* or "five or more" or "5 or more") adj2 (medicat* or medicine? or prescrib* or coprescrib* or co-prescrib* or prescription? or coprescription? or co-prescription? or OTC or "over-the-counter")).ti,kw,kf. (1832)

109 ((excess* or multipl* or "five or more" or "5 or more") adj (drug or drugs)).ti,kw,kf. (5091)

110 (many medication* or many medicine* or many drug?).ti,kw,kf. (206)

111 ((concomitant* or concurrent*) adj2 (medicat* or medicine? or prescrib* or prescription? or OTC or "over-the-counter" or "behind-the-counter")).ti,kw,kf. (1242)

112 ((concomitant* or concurrent*) adj (drug or drugs)).ti,kw,kf. (408)

113 ((omit* or omission?) adj2 (medicat* or medicine? or prescrib* or prescription? or drug or drugs)).ti,kw,kf. (269)

114 ((under$2 or over$2) adj2 (medicat* or prescrib* or prescription?)).ti,kw,kf. (1532)

115 (underprescri* or overprescri*).ti,kw,kf. (702)

116 (quality adj2 (prescrib* or prescription?)).ti,kw,kf. (823)

117 ((multidrug? or multi-drug?) adj2 (pharmacotherap* or pharmaco-therap* or prescrib* or prescription? or regim* or therap* or treatment?)).ti,kw,kf. (3720)

118 (multiple adj (pharmacotherap* or pharmaco-therap*)).ti,kw,kf. (6)

119 ((medication? or medicine? or prescrib* or prescription?) adj3 cascad*).ti,kw,kf. (264)

120 ((medication? or medicine? or prescrib* or prescription?) adj3 continuum*).ti,kw,kf. (69)

121 ((medication? or medicine? or prescrib* or prescription?) adj3 legac*).ti,kw,kf. (116)

122 ((medication? or medicine? or prescrib* or prescription?) adj3 (multi* chronic* or multi* comorbid* or multi* co-morbid* or multimorbid* or multi-morbid*)).ti,kw,kf. (245)

123 (prescrib* adj3 (harm or harmed or harms or harming or harmful*)).ti,kw,kf. (51)

124 potentially inappropriate medication/ (4172)

125 STOPP.ti,kw,kf. (810)

126 "STOPP/START".ti,kw,kf. (422)

127 ((Beers or McLeod or NPS) adj2 criteri*).ti,kw,kf. (970)

128 "Fit fOR The Aged".ti,kw,kf. (97)

129 ((FORTA or RASP or Pricus) adj2 (criteri* or instrument? or list*)).ti,kw,kf. (32)

130 Assessing Care of Vulnerable Elderly.ti,kw,kf. (0)

131 ACOVE.ti,kw,kf. (53)

132 Medication Appropriateness Index.ti,kw,kf. (81)

133 Medication Regimen Complexity.ti,kw,kf. (375)

134 Prescribing Optimi#ation Method.ti,kw,kf. (4)

135 Systematic Tool to Reduce Inappropriate Prescribing.ti,kw,kf. (13)

136 (strip adj5 (medication? or medicine? or prescrib* or prescription?)).ti,kw,kf. (26)

137 or/96-136 [POLYPHARMACY] (52782)

138 adult/ (15631144)

139 exp aged/ (7575928)

140 aged.ti,kw,kf. (224805)

141 ((age? or year?) adj2 ("65" or "66" or "67" or "68" or "69" or "70" or "71" or "72" or "73" or "74" or "75" or "76" or "77" or "78" or "79" or "80" or "81" or "82" or "83" or "84" or "85" or "86" or "87" or "88" or "89" or "90" or "91" or "92" or "93" or "94" or "95" or "96" or "97" or "98" or "99" or "100")).ti,kw,kf. (65924)

142 (elder? or elderly or geriatric* or gerontolog* or old-age? or senior?).ti,kw,kf. (569800)

143 (older adj2 (adult* or female? or male? or man or men or patient? or person? or people? or population? or wom#n)).ti,kw,kf. (324161)

144 *retirement/ (19180)

145 (retiree? or retired or retirement).ti,kw,kf. (23308)

146 (boomer? or babyboomer* or baby-boomer*).ti,kw,kf. (1885)

147 *elderly care/ (22324)

148 exp *geriatric care/ (17631)

149 *home for the aged/ (6749)

150 ((old age or nursing or longterm care or long-term care or LTC) adj (facilit* or home? or residen*)).ti,kw,kf. (70680)

151 or/138-150 [SENIOR FILTER] (19024198)

152 137 and 151 [POLYPHARMACY - SENIOR FILTER] (26547)

153 (exp animal/ or exp animal model/ or exp animal experiment/ or nonhuman/ or exp vertebrate/) not (exp human/ or exp human experiment/) (13491507)

154 152 not 153 [ANIMAL-ONLY REMOVED] (26502)

155 editorial.pt. (1505223)

156 letter.pt. not (letter.pt. and randomized controlled trial/) (2571815)

157 154 not (155 or 156) [OPINION PIECES REMOVED] (25402)

158 (choice? adj2 behavio?r*).ti,kw,kf. (2777)

159 exp *cooperation/ (32222)

160 *decision making/ (194228)

161 *patient decision making/ (2748)

162 *shared decision making/ (5479)

163 decision aid*.ti,kw,kf. (6770)

164 ((guide? or guiding or make or making or makes or made or shar* or support*) adj2 (choice? or choos* or consent* or decid* or decision*)).ti,kw,kf. (192606)

165 *patient education/ (77158)

166 *patient attitude/ (41501)

167 *patient participation/ (31151)

168 *patient preference/ (13645)

169 *patient satisfaction/ (67983)

170 ((engag* or involv* or participat*) adj3 (patient? or person$2 or personally or man or men or "man's" or "men's" or wom#n or "woman's" or "women's")).ti,kw,kf. (38731)

171 *uncertainty/ (23273)

172 ((accept* or consider* or choice? or choos* or chose? or decid* or decis* or expect* or input* or knowledge* or opinion* or participat* or perspective? or prefer* or respons* or satisf* or uncertain* or understand* or willing*) adj2 (female? or male? or man or men or "man's" or "men's" or patient? or person$2 or personally or wom#n or "woman's" or "women's")).ti,kw,kf. (175699)

173 ((analys#s or valuation? or value? or valuing) adj2 (conjoint or contingent)).ti,kw,kf. (3215)

174 (choice? adj1 (discrete or experiment*)).ti,kw,kf. (7591)

175 ((patient? or person$2 or personally or man or men or "man's" or "men's" or wom#n or "woman's" or "women's") adj (centered or centred or focus*)).ti,kw,kf. (46407)

176 ((patient* or person#2) adj priorit*).ti,kw,kf. (974)

177 *informed consent/ (42819)

178 (informed adj (choice* or choos* or consent* or decid* or decision*)).ti,kw,kf. (28758)

179 ((patient? or person$2 or personally or man or men or "man's" or "men's" or wom#n or "woman's" or "women's") adj2 consent*).ti,kw,kf. (1947)

180 ((adher* or nonadher* or non-adher* or inten* or refus* or reject* or uptake or willing*) adj2 (initiat* or intervention? or therap* or treat*)).ti,kw,kf. (51502)

181 (preference? adj1 (elicit* or reveal* or scor* or stated)).ti,kw,kf. (1964)

182 (trade off? or tradeoff? or trade-off?).ti,kw,kf. (21083)

183 exp *information seeking/ (9042)

184 (inform* adj1 seek*).ti,kw,kf. (6294)

185 or/158-184 [PATIENT ACCEPTABILITY/DECISION-MAKING/PARTICIPATION] (901726)

186 157 and 185 [POLYPHARMACY - SENIORS - PATIENT ACCEPTABILITY/DECISION-MAKING/PARTICIPATION] (1064)

187 limit 186 to english (1017)

188 limit 186 to french (5)

189 187 or 188 [LANGUAGE LIMITS APPLIED] (1021)

190 189 use emczd [EMBASE RECORDS] (618)

191 Polypharmacy/ (34493)

192 (polypharm* or poly-pharm*).tw. (37758)

193 (polymedic* or poly-medic*).tw. (2303)

194 (polyprescri* or poly-prescri*).tw. (42)

195 (polypragmas* or poly-pragmas*).tw. (333)

196 (deprescri* or de-prescri*).tw. (6222)

197 ((cancel* or ceas* or cessation? or discontinu* or halt* or stop* or terminat*) adj2 (medicat* or medicine? or prescrib* or prescription?)).tw. (24837)

198 ((cancel* or ceas* or cessation? or discontinu* or halt* or stop* or terminat*) adj (drug or drugs)).tw. (2314)

199 ((appropriate* or inappropriate* or incorrect* or indiscriminat* or unnecessar* or rational* or irrational* or optimal* or optimum or suboptim* or sub-optim*) adj2 (medicat* or medicine? or prescrib* or prescription? or OTC or "over-the-counter" or "behind-the-counter")).tw. (48457)

200 ((appropriate* or inappropriate* or incorrect* or indiscriminat* or unnecessar* or rational* or irrational* or optimal* or optimum or suboptim* or sub-optim*) adj (drug or drugs)).tw. (25455)

201 ((excess* or multipl* or "five or more" or "5 or more") adj2 (medicat* or medicine? or prescrib* or coprescrib* or co-prescrib* or prescription? or coprescription? or co-prescription? or OTC or "over-the-counter")).tw. (17338)

202 ((excess* or multipl* or "five or more" or "5 or more") adj (drug or drugs)).tw. (27090)

203 (many medication* or many medicine* or many drug?).tw. (19508)

204 ((concomitant* or concurrent*) adj2 (medicat* or medicine? or prescrib* or prescription? or OTC or "over-the-counter" or "behind-the-counter")).tw. (22145)

205 ((concomitant* or concurrent*) adj (drug or drugs)).tw. (5154)

206 ((omit* or omission?) adj2 (medicat* or medicine? or prescrib* or prescription? or drug or drugs)).tw. (2199)

207 ((under$2 or over$2) adj2 (medicat* or prescrib* or prescription?)).tw. (24282)

208 (underprescri* or overprescri*).tw. (4465)

209 (quality adj2 (prescrib* or prescription?)).tw. (3146)

210 ((multidrug? or multi-drug?) adj2 (pharmacotherap* or pharmaco-therap* or prescrib* or prescription? or regim* or therap* or treatment?)).tw. (15393)

211 (multiple adj (pharmacotherap* or pharmaco-therap*)).tw. (118)

212 ((medication? or medicine? or prescrib* or prescription?) adj3 cascad*).tw. (493)

213 ((medication? or medicine? or prescrib* or prescription?) adj3 continuum*).tw. (220)

214 ((medication? or medicine? or prescrib* or prescription?) adj3 legac*).tw. (178)

215 ((medication? or medicine? or prescrib* or prescription?) adj3 (multi* chronic* or multi* comorbid* or multi* co-morbid* or multimorbid* or multi-morbid*)).tw. (865)

216 (prescrib* adj3 (harm or harmed or harms or harming or harmful*)).tw. (559)

217 STOPP.tw. (2089)

218 "STOPP/START".tw. (948)

219 ((Beers or McLeod or NPS) adj2 criteri*).tw. (3053)

220 "Fit fOR The Aged".tw. (401)

221 ((FORTA or RASP or Pricus) adj2 (criteri* or instrument? or list*)).tw. (116)

222 Assessing Care of Vulnerable Elderly.tw. (2)

223 ACOVE.tw. (219)

224 Medication Appropriateness Index.tw. (458)

225 Medication Regimen Complexity.tw. (669)

226 Prescribing Optimi#ation Method.tw. (6)

227 Systematic Tool to Reduce Inappropriate Prescribing.tw. (30)

228 (strip adj5 (medication? or medicine? or prescrib* or prescription?)).tw. (93)

229 or/191-228 [POLYPHARMACY] (272279)

230 limit 229 to ("380 aged <age 65 yrs and older>" or "390 very old <age 85 yrs and older>") [Limit not valid in Embase,Ovid MEDLINE(R); records were retained] (261008)

231 aged.ti,id. (205313)

232 ((age? or year?) adj2 ("65" or "66" or "67" or "68" or "69" or "70" or "71" or "72" or "73" or "74" or "75" or "76" or "77" or "78" or "79" or "80" or "81" or "82" or "83" or "84" or "85" or "86" or "87" or "88" or "89" or "90" or "91" or "92" or "93" or "94" or "95" or "96" or "97" or "98" or "99" or "100")).tw. (2616131)

233 (elder? or elderly or geriatric* or gerontolog* or old-age? or senior?).tw. (1216634)

234 (older adj2 (adult* or female? or male? or man or men or patient? or person? or people? or population? or wom#n)).tw. (829091)

235 Retirement/ (37118)

236 (retiree? or retired or retirement).tw. (79747)

237 (boomer? or babyboomer* or baby-boomer*).tw. (6185)

238 exp Elder Care/ (7024)

239 exp Nursing Homes/ (123295)

240 ((old age or nursing or longterm care or long-term care or LTC) adj (facilit* or home? or residen*)).tw. (131658)

241 or/230-240 [SENIOR FILTER] (4483999)

242 229 and 241 [POLYPHARMACY - SENIOR FILTER] (262084)

243 Choice Behavior/ (331139)

244 (choice? adj2 behavio?r*).tw. (15495)

245 Cooperation/ (64644)

246 Decision Making/ (499332)

247 Group Decision Making/ (3766)

248 decision aid*.tw. (12765)

249 ((guide? or guiding or make or making or makes or made or shar* or support*) adj2 (choice? or choos* or consent* or decid* or decision*)).tw. (850724)

250 Client Education/ (4806)

251 Client Participation/ (3413)

252 exp Client Attitudes/ (26618)

253 ((engag* or involv* or participat*) adj3 (patient? or person$2 or personally or man or men or "man's" or "men's" or wom#n or "woman's" or "women's")).tw. (515791)

254 exp Uncertainty/ (85027)

255 ((accept* or consider* or choice? or choos* or chose? or decid* or decis* or expect* or input* or knowledge* or opinion* or participat* or perspective? or prefer* or respons* or satisf* or uncertain* or understand* or willing*) adj2 (female? or male? or man or men or "man's" or "men's" or patient? or person$2 or personally or wom#n or "woman's" or "women's")).tw. (1293514)

256 ((analys#s or valuation? or value? or valuing) adj2 (conjoint or contingent)).tw. (6347)

257 (choice? adj1 (discrete or experiment*)).tw. (15254)

258 ((patient? or person$2 or personally or man or men or "man's" or "men's" or wom#n or "woman's" or "women's") adj (centered or centred or focus*)).tw. (136709)

259 ((patient* or person#2) adj priorit*).tw. (3594)

260 Informed Consent/ (185104)

261 (informed adj (choice* or choos* or consent* or decid* or decision*)).tw. (202763)

262 ((patient? or person$2 or personally or man or men or "man's" or "men's" or wom#n or "woman's" or "women's") adj2 consent*).tw. (51392)

263 ((adher* or nonadher* or non-adher* or inten* or refus* or reject* or uptake or willing*) adj2 (initiat* or intervention? or therap* or treat*)).tw. (383671)

264 (preference? adj1 (elicit* or reveal* or scor* or stated)).tw. (8544)

265 (trade off? or tradeoff? or trade-off?).tw. (102827)

266 Information Seeking/ (9682)

267 (inform* adj1 seek*).tw. (19940)

268 or/243-267 [PATIENT ACCEPTABILITY/DECISION-MAKING/PARTICIPATION] (3604969)

269 242 and 268 [POLYPHARMACY - SENIORS - PATIENT ACCEPTABILITY/DECISION-MAKING/PARTICIPATION] (31508)

270 limit 269 to english (30094)

271 limit 269 to french (285)

272 270 or 271 [LANGUAGE LIMITS APPLIED] (30331)

273 272 use psyh [PSYCINFO RECORDS] (708)

274 95 or 190 or 273 [ALL DATABASES] (7375)

275 limit 274 to yr="2004-current" (6544)

276 limit 275 to yr="2015-current" (4438)

277 remove duplicates from 276 (3864)

278 275 not 276 (2106)

279 remove duplicates from 278 (1874)

280 277 or 279 [**TOTAL UNIQUE RECORDS**] (**5738**)

281 280 use medall [MEDLINE UNIQUE RECORDS] (5281)

282 280 use emczd [EMBASE UNIQUE RECORDS] (238)

283 280 use psyh [PSYCINFO UNIQUE RECORDS] (219)

***************************

Cochrane

ID Search Hits

#1 [mh Polypharmacy] 400

#2 (polypharm* or poly-pharm*):ti,ab,kw 1522

#3 (polymedic* or poly-medic*):ti,ab,kw 108

#4 (polyprescri* or poly-prescri*):ti,ab,kw 2

#5 (polypragmas* or poly-pragmas*):ti,ab,kw 5

#6 [mh Deprescriptions] 109

#7 (deprescri* or de-prescri*):ti,ab,kw 399

#8 ((cancel* or ceas* or cessation* or discontinu* or halt* or stop* or terminat*) NEAR/2 (medicat* or medicine* or prescrib* or prescription*)):ti,ab,kw 2799

#9 ((cancel* or ceas* or cessation* or discontinu* or halt* or stop* or terminat*) NEXT (drug or drugs)):ti,ab,kw 236

#10 [mh "Inappropriate Prescribing"] 282

#11 ((appropriate* or inappropriate* or incorrect* or indiscriminat* or unnecessar* or rational* or irrational* or optimal* or optimum or suboptim* or sub-optim*) NEAR/2 (medicat* or medicine* or prescrib* or prescription* or OTC or "over-the-counter" or "behind-the-counter")):ti,ab,kw 2487

#12 ((appropriate* or inappropriate* or incorrect* or indiscriminat* or unnecessar* or rational* or irrational* or optimal* or optimum or suboptim* or sub-optim*) NEXT (drug or drugs)):ti,ab,kw 1519

#13 ((excess* or multipl* or "five or more" or "5 or more") NEAR/2 (medicat* or medicine* or prescrib* or coprescrib* or co-prescrib* or prescription* or coprescription* or co-prescription* or OTC or "over-the-counter")):ti,ab,kw 728

#14 ((excess* or multipl* or "five or more" or "5 or more") NEXT (drug or drugs)):ti,ab,kw 2499

#15 (many NEXT (medication* or medicine* or drug or drugs)):ti,ab,kw 475

#16 ((concomitant* or concurrent*) NEAR/2 (medicat* or medicine* or prescrib* or prescription* or OTC or "over-the-counter" or "behind-the-counter")):ti,ab,kw 3542

#17 ((concomitant* or concurrent*) NEXT (drug or drugs)):ti,ab,kw 310

#18 ((omit* or omission*) NEAR/2 (medicat* or medicine* or prescrib* or prescription* or drug or drugs)):ti,ab,kw 99

#19 ((under or over or overly) NEAR/2 (medicat* or prescrib* or prescription*)):ti,ab,kw 1948

#20 (underprescri* or overprescri*):ti,ab,kw 249

#21 (quality NEAR/2 (prescrib* or prescription*)):ti,ab,kw 478

#22 ((multidrug* or multi-drug*) NEAR/2 (pharmacotherap* or pharmaco-therap* or prescrib* or prescription* or regim* or therap* or treatment*)):ti,ab,kw 812

#23 (multiple NEXT (pharmacotherap* or pharmaco-therap*)):ti,ab,kw 7

#24 ((medication* or medicine* or prescrib* or prescription?) NEAR/3 cascad*):ti,ab,kw 6

#25 ((medication* or medicine* or prescrib* or prescription?) NEAR/3 continuum*):ti,ab,kw 2

#26 ((medication* or medicine* or prescrib* or prescription?) NEAR/3 legac*):ti,ab,kw 0

#27 ((medication* or medicine* or prescrib* or prescription?) NEAR/3 (multi* chronic or multi* comorbid* or multi* co-morbid* or multimorbid* or multi-morbid*)):ti,ab,kw 4973

#28 (prescrib* NEAR/3 (harm or harmed or harms or harming or harmful*)):ti,ab,kw 34

#29 [mh "Potentially Inappropriate Medication List"] 74

#30 STOPP:ti,ab,kw 170

#31 "STOPP/START":ti,ab,kw 74

#32 ((Beers or McLeod or NPS) NEAR/2 criteri*):ti,ab,kw 74

#33 "Fit fOR The Aged":ti,ab,kw 5

#34 ((FORTA or RASP or Pricus) NEAR/2 (criteri* or instrument* or list*)):ti,ab,kw 22

#35 "Assessing Care of Vulnerable Elderly":ti,ab,kw 0

#36 ACOVE:ti,ab,kw 10

#37 "Medication Appropriateness Index":ti,ab,kw 79

#38 "Medication Regimen Complexity":ti,ab,kw 29

#39 ("Prescribing Optimisation Method" or "Prescribing Optimization Method"):ti,ab,kw 1

#40 "Systematic Tool to Reduce Inappropriate Prescribing":ti,ab,kw 6

#41 (strip NEAR/5 (medication* or medicine* or prescrib* or prescription*)):ti,ab,kw 11

#42 {or #1-#41} 22300

#43 [mh ^Adult] 434959

#44 [mh Aged] 275454

#45 aged:ti,kw 540302

#46 ((age or aged or ages or year or years) NEAR/2 ("65" or "66" or "67" or "68" or "69" or "70" or "71" or "72" or "73" or "74" or "75" or "76" or "77" or "78" or "79" or "80" or "81" or "82" or "83" or "84" or "85" or "86" or "87" or "88" or "89" or "90" or "91" or "92" or "93" or "94" or "95" or "96" or "97" or "98" or "99" or "100")):ti,ab,kw 247759

#47 (elder or elders or elderly or geriatric* or gerontolog* or "old-age" or "old-aged" or senior or seniors):ti,ab,kw 134565

#48 (older NEAR/2 (adult* or female or females or male or males or man or men or patient or patients or person or persons or people or peoples or population* or woman or women)):ti,ab,kw 41046

#49 [mh Retirement] 101

#50 (retiree* or retired or retirement):ti,ab,kw 1050

#51 (boomer* or babyboomer* or baby-boomer*):ti,ab,kw 84

#52 [mh "Health Services for the Aged"] 580

#53 [mh "Homes for the Aged"] 824

#54 (("old age" or nursing or "longterm care" or "long-term care" or LTC) NEXT (facilit* or home or homes or residen*)):ti,ab,kw 6260

#55 {or #43-#54} 850775

#56 #42 and #55 10558

#57 [mh "Choice Behavior"] 1982

#58 (choice* NEAR/2 behavio*):ti,ab,kw 1960

#59 [mh "Cooperative Behavior"] 1200

#60 [mh ^"Decision Making"] 3425

#61 [mh "Decision Making, Shared"] 189

#62 (decision NEXT aid*):ti,ab,kw 1817

#63 ((guide or guided or guides or guiding or make or making or makes or made or shar* or support*) NEAR/2 (choice* or choos* or consent* or decid* or decision*)):ti,ab,kw 27630

#64 [mh "Patient Education as Topic"] 10867

#65 [mh "Patient Acceptance of Health Care"] 23269

#66 [mh "Patient Participation"] 2195

#67 [mh "Patient Preference"] 1204

#68 [mh "Patient Satisfaction"] 15517

#69 ((engag* or involv* or participat*) NEAR/3 (patient or patients or person or personal or personally or man or men or "man's" or "men's" or woman or women or "woman's" or "women's")):ti,ab,kw 42362

#70 [mh Patients/PX] 874

#71 [mh Uncertainty] 386

#72 ((accept* or consider* or choice* or choos* or chose or chosen or decid* or decis* or expect* or input* or knowledge* or opinion* or participat* or perspective* or prefer* or respons* or satisf* or uncertain* or understand* or willing*) NEAR/2 (female or females or male or males or man or men or "man's" or "men's" or patient or patients or person or personal or personally or woman or women or "woman's" or "women's")):ti,ab,kw 127642

#73 ((analysis or analyses or valuation* or value or valued or values or valuing) NEAR/2 (conjoint or contingent)):ti,ab,kw 147

#74 (choice* NEAR/1 (discrete or experiment*)):ti,ab,kw 397

#75 ((patient or patients or person or personal or personally or man or men or "man's" or "men's" or woman or women or "woman's" or "women's") NEXT (centered or centred or focus*)):ti,ab,kw 6992

#76 ((patient or patients or person or personal) NEXT priorit*):ti,ab,kw 304

#77 [mh "Informed Consent"] 1478

#78 (informed NEXT (choice* or choos* or consent* or decid* or decision*)):ti,ab,kw 100607

#79 ((patient or patients or person or personal or personally or man or men or "man's" or "men's" or woman or women or "woman's" or "women's") NEAR/2 consent*):ti,ab,kw 15603

#80 ((adher* or nonadher* or non-adher* or inten* or refus* or reject* or uptake or willing*) NEAR/2 (initiat* or intervention* or therap* or treat*)):ti,ab,kw 81739

#81 (preference* NEAR/1 (elicit* or reveal* or scor* or stated)):ti,ab,kw 337

#82 ("trade off" or "trade offs" or tradeoff* or trade-off*):ti,ab,kw 1167

#83 [mh "Information Seeking Behavior"] 78

#84 (inform* NEAR/1 seek*):ti,ab,kw 440

#85 {or #57-#84} 350112

#86 #56 AND #85 3575

#87 #56 AND #85 with Cochrane Library publication date Between Jan 2004 and Dec 2024, in Cochrane Reviews, Cochrane Protocols 59

**eTable 1. Examples of excluded studies**

| **Study** | **Reason for exclusion** |
| --- | --- |
| Jungo KT, Deml MJ, Schalbetter F, Moor J, Feller M, Lüthold RV, Huibers CJA, Sallevelt BTGM, Meulendijk MC, Spruit M, Schwenkglenks M, Rodondi N, Streit S. A mixed methods analysis of the medication review intervention centered around the use of the 'Systematic Tool to Reduce Inappropriate Prescribing' Assistant (STRIPA) in Swiss primary care practices. BMC Health Serv Res. 2024 Mar 18;24(1):350. doi: 10.1186/s12913-024-10773-y. Erratum in: BMC Health Serv Res. 2024 Apr 2;24(1):413. doi: 10.1186/s12913-024-10896-2. PMID: 38500163; PMCID: PMC10949561. | Qualitative methods were used to measure acceptability outcomes. |
| Moga DC, Abner EL, Rigsby DN, Eckmann L, Huffmyer M, Murphy RR, Coy BB, Jicha GA. Optimizing medication appropriateness in older adults: a randomized clinical interventional trial to decrease anticholinergic burden. Alzheimers Res Ther. 2017 May 23;9(1):36. doi: 10.1186/s13195-017-0263-9. PMID: 28535785; PMCID: PMC5442667. | Acceptability outcomes lacked a comparator. |
| Martin P, Tamblyn R, Benedetti A, Ahmed S, Tannenbaum C. Effect of a Pharmacist-Led Educational Intervention on Inappropriate Medication Prescriptions in Older Adults: The D-PRESCRIBE Randomized Clinical Trial. JAMA. 2018 Nov 13;320(18):1889-1898. doi: 10.1001/jama.2018.16131. PMID: 30422193; PMCID: PMC6248132. | Acceptability outcomes were not compared between groups. |
| Reeve E, Andrews JM, Wiese MD, Hendrix I, Roberts MS, Shakib S. Feasibility of a patient-centered deprescribing process to reduce inappropriate use of proton pump inhibitors. Ann Pharmacother. 2015 Jan;49(1):29-38. doi: 10.1177/1060028014558290. Epub 2014 Nov 10. PMID: 25385826. | Effectiveness study conducted without a comparator group. |
| Rieckert A, Teichmann AL, Drewelow E, Kriechmayr C, Piccoliori G, Woodham A, Sönnichsen A. Reduction of inappropriate medication in older populations by electronic decision support (the PRIMA-eDS project): a survey of general practitioners' experiences. J Am Med Inform Assoc. 2019 Nov 1;26(11):1323-1332. doi: 10.1093/jamia/ocz104. PMID: 31504572; PMCID: PMC6798559. | Only the intervention arm was included in the study. |
| van der Meer HG, Wouters H, Teichert M, Griens F, Pavlovic J, Pont LG, Taxis K. Feasibility, acceptability and potential effectiveness of an information technology-based, pharmacist-led intervention to prevent an increase in anticholinergic and sedative load among older community-dwelling individuals. Ther Adv Drug Saf. 2018 Oct 30;10:2042098618805881. doi: 10.1177/2042098618805881. PMID: 31019675; PMCID: PMC6463339. | Acceptability outcomes lacked a comparator. |

**List of included studies**

1. Alaa Eddine N, Schreiber J, El-Yazbi AF, Shmaytilli H, Amin MEK. A pharmacist-led medication review service with a deprescribing focus guided by implementation science. *Front Pharmacol.*2023;14:1097238. Published 2023 Jan 30. doi:10.3389/fphar.2023.1097238
2. Community Pharmacy Medicines Management Project Evaluation Team. The MEDMAN study: a randomized controlled trial of community pharmacy-led medicines management for patients with coronary heart disease. *Fam Pract.* 2007;24(2):189-200. doi:10.1093/fampra/cml075
3. Del Cura-González I, López-Rodríguez JA, Leiva-Fernández F, et al. How to Improve Healthcare for Patients with Multimorbidity and Polypharmacy in Primary Care: A Pragmatic Cluster-Randomized Clinical Trial of the MULTIPAP Intervention. *J Pers Med*. 2022;12(5):752. Published 2022 May 6. doi:10.3390/jpm12050752
4. Hamilton W, Russell D, Stabb C, Seamark D, Campion-Smith C, Britten N. The effect of patient self-completion agenda forms on prescribing and adherence in general practice: a randomized controlled trial. *Fam Pract.* 2007;24(1):77-83. doi:10.1093/fampra/cml057
5. Hugtenburg JG, Borgsteede SD, Beckeringh JJ. Medication review and patient counselling at discharge from the hospital by community pharmacists. *Pharm World Sci.* 2009;31(6):630-637. doi:10.1007/s11096-009-9314-z
6. Linsky AM, Kressin NR, Stolzmann K, et al. Direct-to-consumer strategies to promote deprescribing in primary care: a pilot study. *BMC Prim Care.* 2022;23(1):53. Published 2022 Mar 22. doi:10.1186/s12875-022-01655-5
7. McCarthy C, Clyne B, Boland F, et al. GP-delivered medication review of polypharmacy, deprescribing, and patient priorities in older people with multimorbidity in Irish primary care (SPPiRE Study): A cluster randomised controlled trial. *PLoS Med.* 2022;19(1):e1003862. Published 2022 Jan 5. doi:10.1371/journal.pmed.1003862
8. Mecca MC, Zenoni M, Fried TR. Primary care clinicians' use of deprescribing recommendations: A mixed-methods study. *Patient Educ Couns.* 2022;105(8):2715-2720. doi:10.1016/j.pec.2022.04.013
9. Messerli M, Vriends N, Hersberger KE. Humanistic outcomes and patient acceptance of the pharmacist-led medication review "Polymedication Check" in primary care in Switzerland: a prospective randomized controlled trial. *Patient Prefer Adherence.*2018;12:1071-1078. Published 2018 Jun 19. doi:10.2147/PPA.S160789

**eFigure 1. Summary of risk of bias assessments for included randomized studies (ROB-2)**


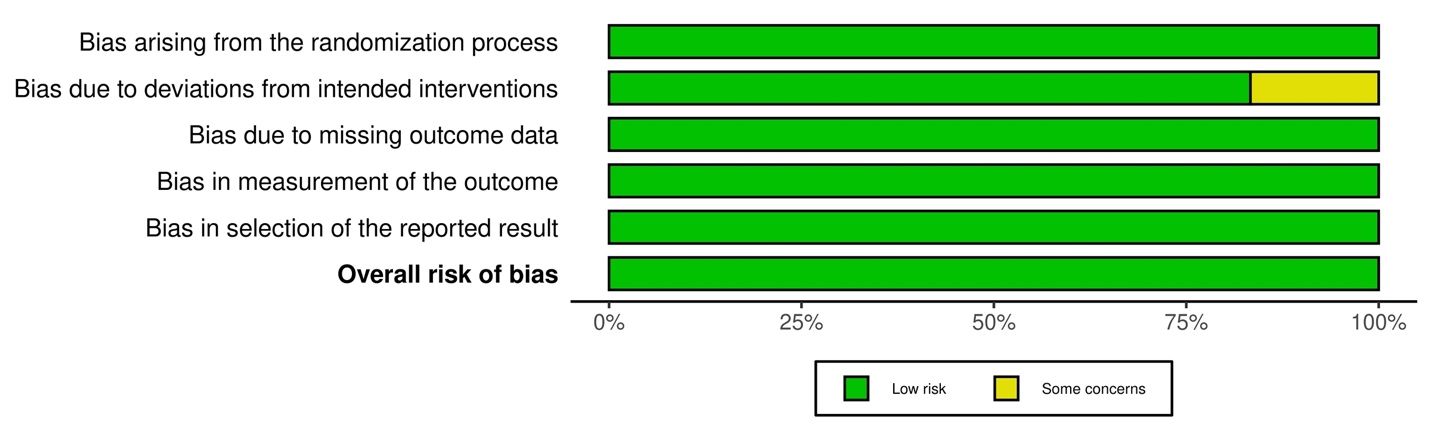


**eFigure 2. Risk of bias assessment for each included randomized trial (ROB-2)**


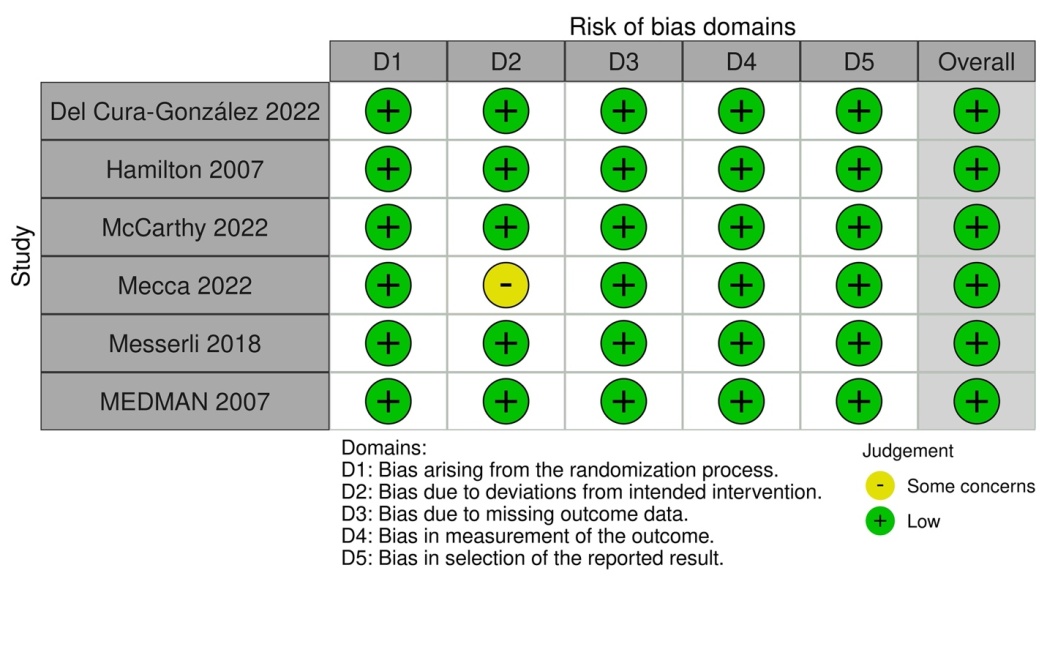


**eFigure 3. Summary of risk of bias assessments for included non-randomized studies(ROBINS-I)**


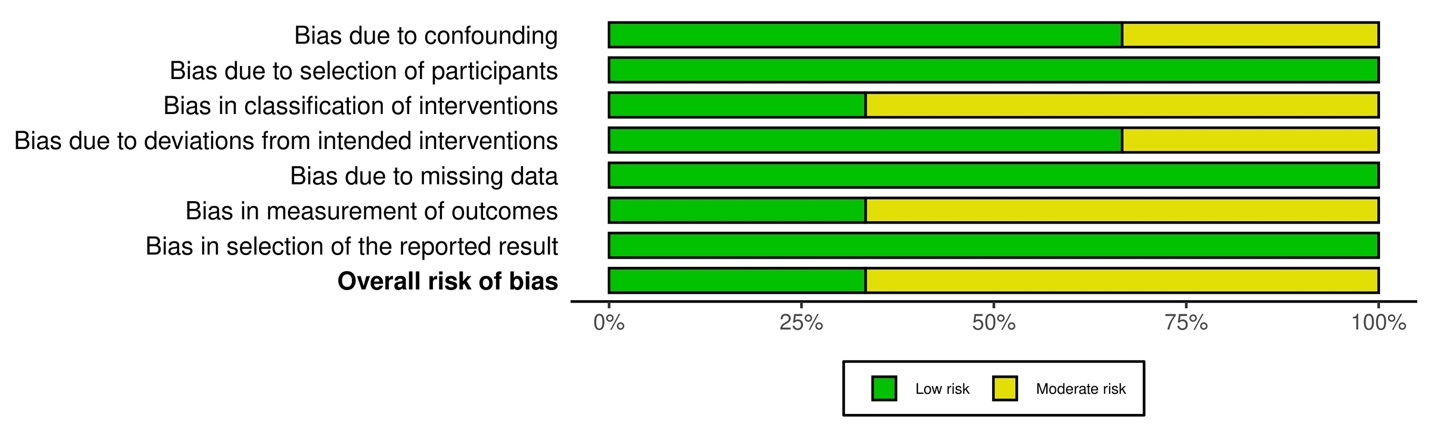


**eFigure 4. Risk of bias assessment for each included non-randomized trial (ROBINS-I)**


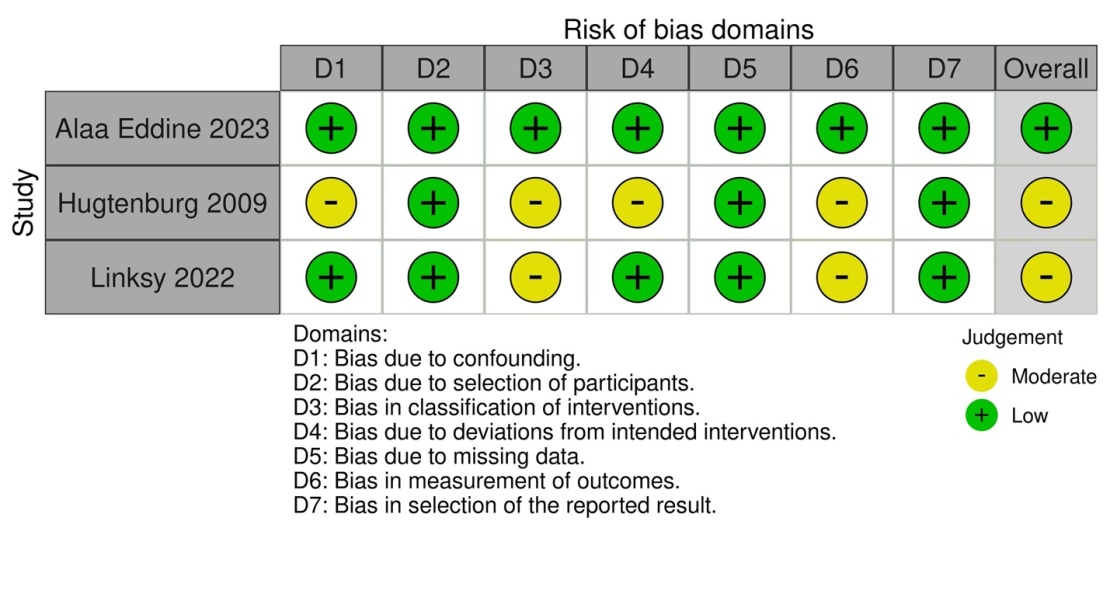


**eFigure 5. Funnel plot assessing publication bias in studies reporting patient satisfaction with PIP interventions versus usual care**
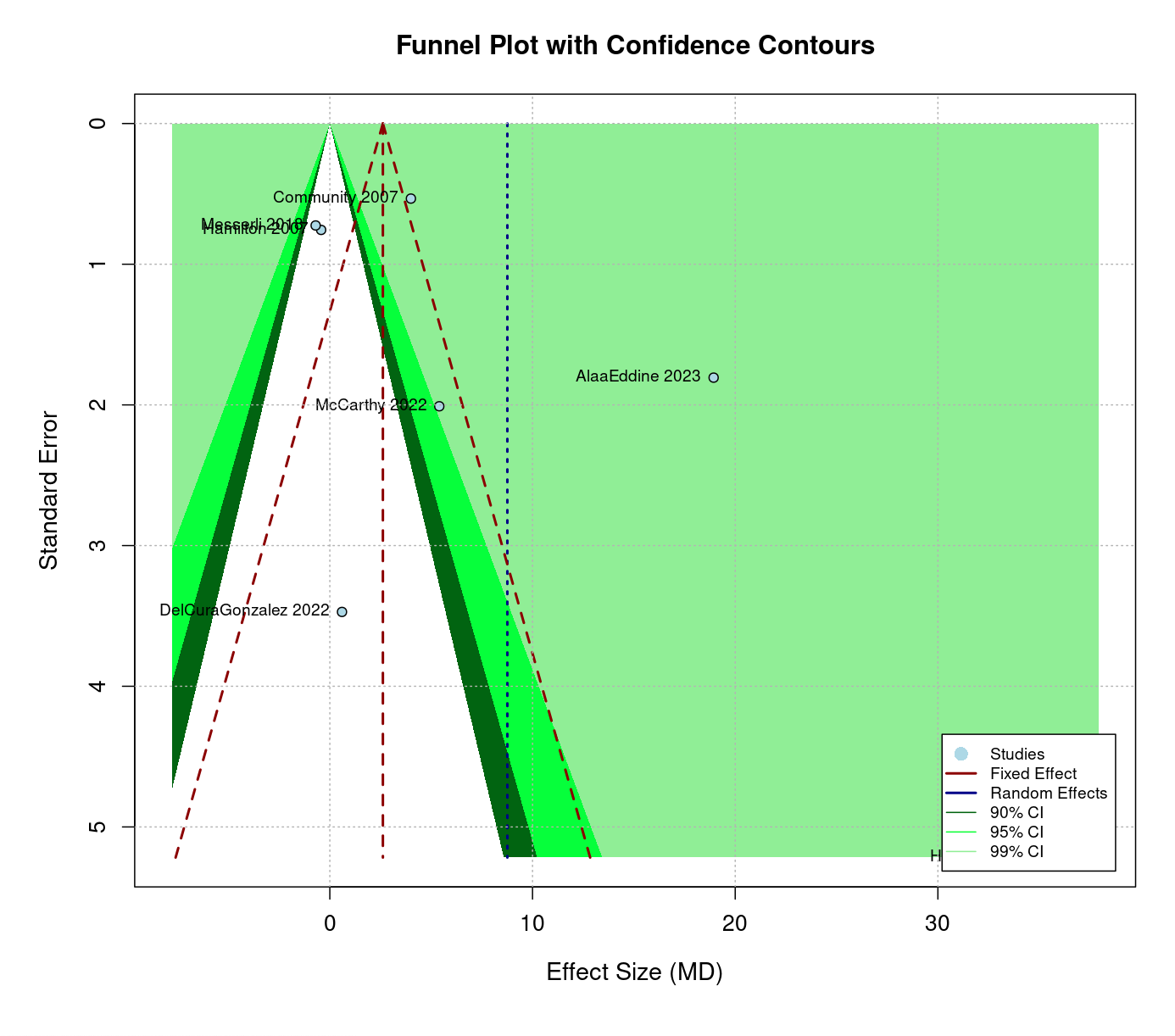

Supplement: Supplementary file 1 — PIPacceptabilityAppendix2025 04 04. [file HSR2-8-e70981-s001.docx]
